# Family before work: task reversion in workers of the red imported fire ant, *Solenopsis invicta* in the presence of brood

**DOI:** 10.1038/s41598-023-29246-z

**Published:** 2023-02-10

**Authors:** Jesse Starkey, Cecilia Tamborindeguy

**Affiliations:** Department of Entomology, 412 Heep Center, Texas A&M University, College Station, TX 77843 USA

**Keywords:** Social evolution, Behavioural ecology

## Abstract

Among social insects, task allocation within its group members remains as one of the paramount pillars of social functionality. Division of labor in many eusocial insects is maintained by behavioral flexibility that can shift according to the needs of the colony they reside in. Workers typically, over time as they age, shift from intranidal nurses to extranidal foragers. If the needs of the colony change, either from the needs of the adults or the brood therein, workers shift their behavior in order to compensate for the need of a particular task to be done. This shift, either accelerating towards a behavior associated with an older worker, or regressing back into the nest, is not clearly understood in social insects outside of honeybees. In this study, evaluated how brood type affected the red imported fire ant, *Solenopsis invicta,* worker task reversion and acceleration. Through observation of worker behaviors performed over multiple time-points per day, we discovered that worker task reversion and acceleration does occur within this ant species. Furthermore, the type of brood influenced the rate at which this occurred, with larvae having the strongest effect of all types. Finally, there was a propensity for workers to maintain their new behavior throughout the experiment. This study shows that the needs of brood within a social insect colony can influence the behavior workers perform, reversing the age polyethism that is common among social insect species.

## Introduction

Division of labor is a central pillar of eusociality in which tasks are divided among reproductive and non-reproductive castes within a group of organisms^1^. Tasks within the caste of non-reproductive individuals can be incredibly varied and are often associated with the age of the individual. Younger workers have a tendency to stay within the nest and tend to the queen and brood, while older workers tend to leave the nest and perform extranidal behaviors such as guarding and foraging^2,3^.

Since sociality has evolved multiple times, each bearing its roots in simple family structure where adults care for young, it would be likely that caring for young could affect changes in worker polyethism. In many eusocial insects, young brood are helpless and require intensive care from adult workers to survive; examples of care include feeding, cleaning, and thermoregulation^4,5^. The method by which the brood communicate their need to adults varies based on the organism, utilizing chemical communication, either volatile or contact, to convey their need to nearby workers, as well as implementing physical begging behaviors in order to cause a shift in behavior of workers either accelerating towards performing tasks characteristic of older workers, or reverting the behavior of older workers to that of a younger worker^6–9^. If task reversion occurs, that is older workers performing tasks associated with older insects switch back to perform younger worker type tasks, it must be regulated by a similar mechanism that gauges the needs of the colony.

The fixed response threshold has the potential to align with the idea that brood can change the behavior among workers and ultimately influence division of labor within an eusocial insect colony^10^. The model is described as an interaction between task-related stimuli and internal thresholds, where workers performing a certain task reduce the stimuli which result in less recruitment of workers to begin performing the task, thus reducing the number of workers recruited. As the brood begins to beg or communicate their need to adult workers, the stimuli to either be recruited for nursing or foraging will increase based on the brood’s need, and thus allow for reorganization of division of labor within the worker caste of insects.

Division of labor among workers via age-related polyethism has been shown to change based on the needs of the colony. An example of this can be found in *Phedole dentata* colonies where when a shortage of young workers is detected, older workers will perform the tasks typically done by young workers as well as their usual tasks. This also happens in many other social insect species^11–14^. Task reversion has been prominently studied in the honeybee, *Apis mellifera*, where when nurses are removed from the colony, foragers switch back to nurses. While this is ultimately a behavioral shift in the worker, physiological changes could also occur such as titers of juvenile hormone^13,15^, which decrease in levels to those of a nurse, as well as increasing the amount of DNA methylation to be on par with nurses^16^.

While task reversion has been prominently studied in honeybees, it occurs in other eusocial Hymenoptera as well. Following colony manipulation via removing some brood and nurse workers, foragers of the ant genus *Diacamma* reverted into nurses at a high rate^17–19^. Other eusocial insects such as *Platythyrea punctata*, *Temnothorax albipennis*, and *Pheidole dentata*^11,20–22^ have also been shown to display task reversion from foragers to nurses as a result of colony change and needs, as such behavior appears to be particularly prevalent in social systems where worker polyethism is present. Similarly, acceleration of task progression can also be accomplished via changes in gene expression. For example, experimental knockdown of *Vg-like* gene expression in nurses caused a shift in behavior from brood care to nestmate care, a characteristic representative of older workers in the ant *Temnothorax longispinosus*^23^.

There have also been a series of identified genes that, within eusocial insect species, have been characterized and can be used as markers to differentiate castes within these species. Hexamerins are a family of storage proteins most notably known for being larval storage proteins that are synthesized in the fat body by larvae and reabsorbed during pupation^24^. Hexamerins have also been shown to be associated with social organization and behavior; silencing of these genes resulted in the development of soldier-caste workers in termites^25,26^. Additionally, differential expression of hexamerins was found between members of the worker castes performing different tasks in many eusocial insects^27–30^. Anti-microbial peptides (AMPs), a major line of defense for insect immunity were also identified as quality biomarkers for different castes in social insects, likely due to social immunity^31,32^. For instance, numerous AMPs such as *abaecin* and *apidaecin* are upregulated in forager honey bee workers compared to nurses^33^ and AMPs like *defensin* and *hymenoptaecin* are differentially expressed between foragers and nurses in the leaf-cutting ant *Atta vollenweideri*^34^. Expression of the *foraging* gene has been shown to be involved with foraging activity^35^, olfaction^36^, learning^37^, and stress tolerance^38^. Within the scope of social insects, the *foraging* gene is associated with labor division among worker castes in ants ^39–41^ as well as bees and wasps^42,43^.

The red imported fire ant, *Solenopsis invicta*, is an invasive ant species in the United States, Caribbean, Australia, as well as Southeastern and Eastern Asia^44,45^. Colonies can either be polygyne or monogyne, being headed by multiple queens or a single queen, respectively. Monogyne workers are larger^46^ and behaviorally more aggressive than polygyne workers^47^, but polygyne colonies typically are larger than monogyne colonies due to the creation of supercolonies^48^ among numerous other characteristics. Workers exhibit age polyethism where younger workers remain in the nest to tend to the queen and brood, and older workers leave the nest and perform tasks such as nest defense and foraging^49^. This change in behavior as the worker ages is also associated with the subcaste type: minor, medium, or major, where the larger majors leave the nest sooner than the smaller minors^50–52^. Workers have also been shown to shift task performed within the colony based on the needs and stimuli received from the colony itself such as food^53^. Additionally, there is evidence that the brood produces a volatile pheromone that influences brood tending behavior in workers^54^. Furthermore, the presence of brood in *S. invicta* influences expression of the short neuropeptide F receptor, a receptor of a neuropeptide of the same name that is involved in olfaction and sub-esophageal ganglion functions. Different localization of the receptor was observed in the brain of workers depending the presence or absence of brood in the colonies^55–57^. The interplay between brood and adults in eusocial and social insects is difficult to ignore considering their proximity to one another within the colony, in addition to the frequency by which these two groups interact with one another. These interactions are even more important in *S. invicta,* as 4th instar larvae are required to process solid protein within the colony. Brood has also been shown to influence worker foraging behavior determined by the needs of the brood themselves^58–62^.

In this study, we aimed to determine the effect of brood on task reversion in *S. invicta*, particularly when brood type is manipulated. We hypothesized that in the absence of nurses, foragers would return to the nest to tend to the brood and that larvae would have a stronger effect on workers than eggs or pupae, resulting in a higher proportion of foragers returning to the nest to tend to the brood. We also hypothesized that nurses in the absence of foragers would forage to acquire nutrients for the brood. Further, we aimed to observe if workers recruited from the foraging arena to the nest or accelerated behaviorally from the nest to the foraging arena were consistently the same workers or not. We examined the interplay between brood and worker task regression and acceleration in this invasive ant.

## Materials and Methods

### Fire ant colony maintenance

Colonies of polygyne *S. invicta* were collected in College Station, Brazos County, Texas from March to June of 2021 by digging nests with brood, queens, and workers from the colony on-site and carrying them to the lab in buckets. Then, the colonies were dripped to remove the ants from the collected substrate^63,64^ and maintained in a temperature-controlled room in the Minnie Belle Heep Center, Texas A&M University, College Station, Texas. Colonies were reared and maintained in plastic containers (27 × 40 × 9 cm) coated with Fluon (Insect-a-slip, Bioquip Products, CA) to prevent ants from escaping the container. The room was kept at around 27 °C at a 12:12 h dark–light photoperiod. Colonies were provided with ample supplies of 20% honey solution as well as crickets (*Acheta domestica*) for their source of protein. Water was provided ad libitum in glass test tubes stopped with cotton. Large petri dishes (15 × 5 cm) were provided to each colony as a nesting site. All colonies used contained a single dealate queen, males, multiple unmated alate queens, as well as workers and various brood types (eggs, larvae, pupae). Workers were taken from colonies and DNA as extracted followed by a multiplex PCR as described in Valles and Porter ^65^ in order to confirm that these colonies were indeed polygyne. Polygyne queens were used because they are the most common type in Brazos County, Texas^46^.

### Classification of ant workers and brood

Workers were classified based on their head size^49^. Only medium-sized workers were used (head width between 0.73 and 0.92 mm) for the experiments described here. Foraging workers were determined as workers directly interacting with the honey water or the protein sources that were provided to them outside the nest, whereas nurses were determined via active interaction with any of the brood types (i.e. antennation, mandibular prodding, carrying, or feeding of the brood). The age of individual ants was not tracked. All individual workers used in the bioassays were uniquely marked on the gaster and pronotum using Testors Enamel Paints (Testors, Vernon Hills, Illinois). Eggs, larvae, and pupae were identified and collected for this experiment as well. Newly-developed pupae were determined based on the melanization of the cuticle, and 4th instar larvae were determined via the presence of sclerotized mandibles^62^.

### Task reversion assay

Medium nurses and foragers were collected and marked as previously described. Micro-colonies were set up in a 10 × 10 × 30 cm plastic container coated with fluon. On one end of the box, 20% honey water-soaked cotton as well as crickets were placed ad libitum, whereas the other side had cotton saturated with autoclaved water in addition to a small petri dish (2.5 × 4 cm) with 0.015 g of brood (eggs, larvae, or pupae). Three separate microcolonies were set up with 0.015 g of either eggs, 4th instar larvae, or pupae present within the petri dish nest. Workers were then added to the micro-colonies; 10 marked foragers and 10 marked nurses were added to each colony. After a 24-h acclimation period, one of the microcolonies had all their nurses removed, one had all their foragers removed, and one had none of their workers removed (control). This was considered time 0. After the removal of either the foragers or nurses, the microcolonies were observed 5 times, tallying the number of workers present either tending to the brood or interacting with the food sources every 5 min, for an observation period of 25 min (time points 1, 2, 3, 4, and 5 corresponding to 5, 10, 15, 20, and 25 min respectively). This was considered day 1. The observations were repeated 24 and 48 h later, i.e., days 2 and 3, respectively. Workers were considered performing nurse-like tasks if they were in the vicinity of the nest or directly interacting with the brood, and workers were considered to be performing forager-like tasks if they were directly interacting with any of the food sources, carbohydrate or protein, or present within the foraging arena in general. In addition, the markings of the workers were used to track which individuals at a given time were present either performing nest-like or foraging-like tasks. Each treatment group was replicated 4 times. At the end of the experiment, ants from each bioassay were collected and pooled according to the task being performed on the last observation, snap frozen in liquid nitrogen and stored at − 80 °C until further analyses.

### Quantitative RT-PCR

RNA was extracted from the pools of workers of each different treatment using the OMNI RNA Tissue Purification Kit (OMNI International, Kennesaw, Georgia). cDNA was synthesized using a Verso cDNA Synthesis Kit (ThermoFisher Scientific, Waltham, Massachusetts) along with 250 ng of RNA from each sample. Each reaction for qPCR contained 5µL PowerUp Sybr master mix, primers (10 mM final concentration, Table S1), as well as 10 ng of cDNA. RT-qPCR was performed using the Applied Biosystems QuantStudio 6 Flex Real-Time PCR System (ThermoFisher Scientific). Each reaction was performed in duplicate, and negative controls were included in each plate. Expression of each gene was calculated using the ΔΔCt method^66^ by normalizing the expression of each gene to the housekeeping gene *rpl18,* which is stable among various castes of *S. invicta*^67^. The expression of workers in the nest in the control treatment in the presence of eggs was used as the reference group. Tested genes were *hexamerin* (hereby referred to as *hex2*), *hymenoptaecin* and the *S. invicta foraging* gene (hereby referred to as *sifor*) because those were shown to be differentially expressed between foragers and nurses have different expression profiles: *hymenoptaecin* and *sifor* are both up-regulated in foragers compared to nurses within *S. invicta*, the opposite can be seen for *hex2*^27,68,69^ . The expression profile of these genes was verified using nurses and foragers from the laboratory colonies before the selection of workers for the micro-colonies.

### Statistical analyses

The data reported here are the mean ± the standard error of the mean (SEM). Normality of data was determined via a Chi-squared goodness-of-fit test. Hereafter, treatment will be considered each of the worker manipulation: “nurses” correspond to colonies in which all foragers were removed, “foragers” correspond to colonies in which all nurses were removed and control represents colonies were workers were not removed. Brood type refers to the brood present in the micro-colonies (eggs, larvae and pupae). Days refer to the day of the observation (day 1, 2 and 3). Time-point will refer to each of the 5 daily observation time points.

For the time point reversion analysis, for each day and brood type independently, the proportion of workers performing nest-like, or forager-like behavior was analyzed using a Multivariate ANOVA (MANOVA) with treatment and time point within the day as factors. Time point 0 was not included in the statistical analysis.

For the task reversion analysis, each day and for each brood type the proportion of ants (ranging from 0 to 1) performing nest-like or foraging-like behaviors at each time point was averaged. Data was analyzed for each treatment using a One-way ANOVA followed by a Post-Hoc Tukey HSD test in JMP Pro 16. *P*-values of less than 0.05 were considered significant.

For the worker tracking analysis, for each day and brood type, the task performed by each ant was compared between time point 1 and 5. A contingency table was created and analyzed through a Contingency Analysis in JMP Pro 16. *P*-values less than 0.05 were considered significant.

Differences in gene expression between different treatments were determined via a generalized linear mixed model with *P*-values of less than 0.05 being considered significant. When we did not observe any significance due to an increased amount of variation from this experiment, a one-way ANOVA was used to determine significance in gene expression between treatments, separated by brood types. *P*-values of less than 0.05 were considered significant for this experiment as well.

## Results

### Time point reversion analysis

**Figure 1:**
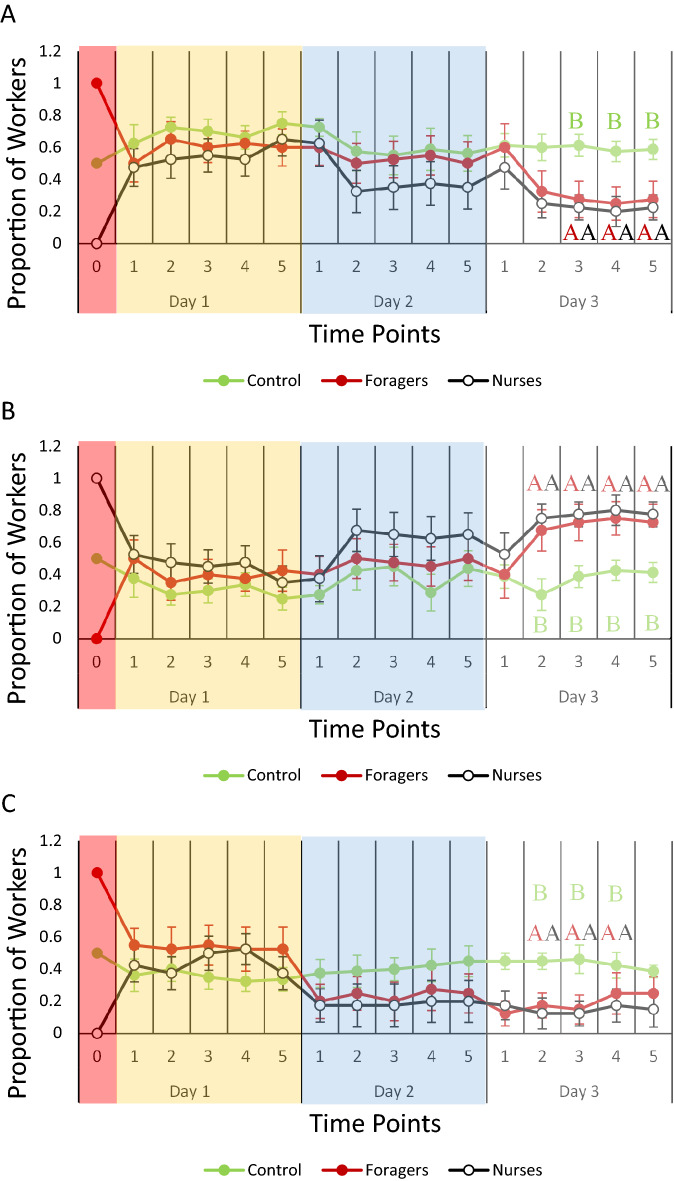

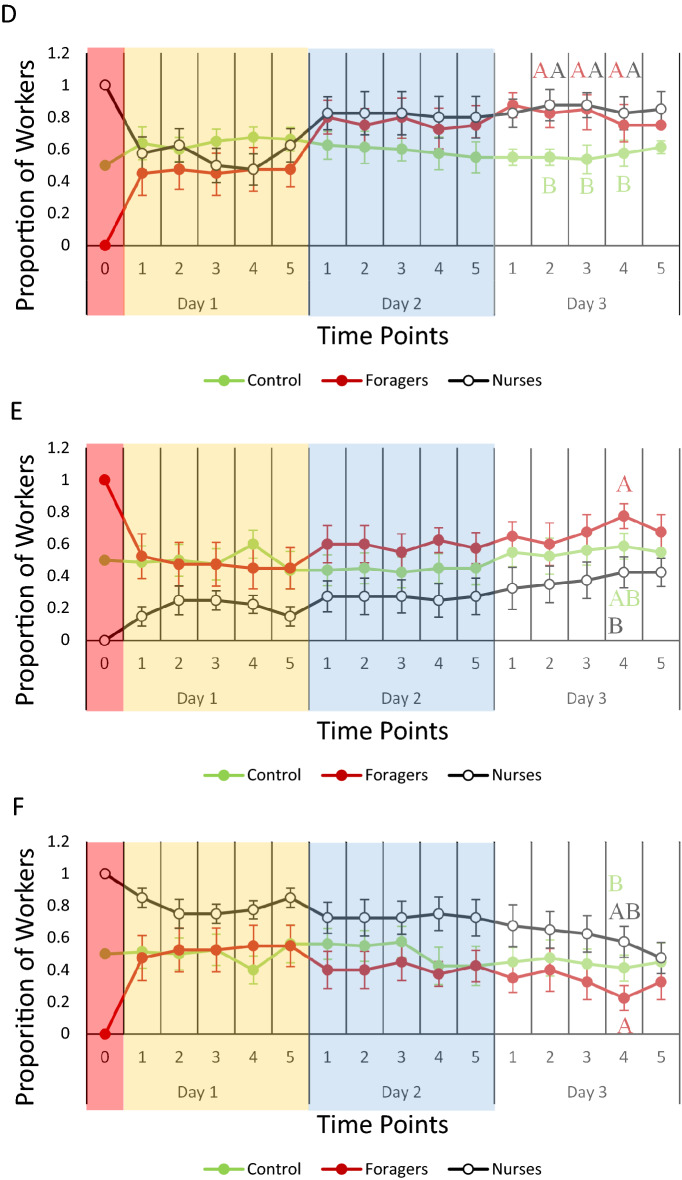
Time point reversion analysis: average proportion (± standard error of the mean) of workers from each treatment group found in different positions within the microcolony during the 48-h observation period. Graphs show the average proportion of workers found (**A**) in the foraging arena and (**B**) in the nest in the presence of eggs; (**C**) in the foraging arena and (**D**) in the nest in the presence of larvae; and (**E**) in the foraging arena and (**F**) in the nest in the presence of pupae. Colored boxes indicate the different days, and each number within each day represents a single observation time point. Time point 0 represents the time before the experiment began, when the type of worker in the micro-colony was manipulated. Different letters indicate statistically significant differences among to each group (*p* < 0.05), and colors correspond with each treatment group. Time point 0 was not included in the statistical analysis.

Each brood type significantly affected the number of workers that reverted or accelerated into another task within the micro colonies. Each day, workers were observed moving between and within either the foraging or nursing areas within the microcolonies (Fig. 1). The time point did not have a significant effect on the proportion of workers found either in the nest or in the foraging arena for workers in the presence of eggs, larvae, and pupae during the duration of the experiment (Table S3A).

Eggs had a significant effect on the proportion of workers found either in the foraging arena (Fig. 1A) from time points 3 to 5and in the nest (Fig. 1B) from time points 2 to 5 for day 3 (Table S3B). The proportion of workers found in the foraging arena in both nurse-only and forager-only treatments were significantly lower when compared to controls at day 3 at time point 3 (Forager-only: *p* = 0.0179; Nurse-only: *p* = 0.0083), 4 (Forager-only: *p* = 0.0188; Nurse-only: *p* = 0.0085), and 5 (Forager-only: *p* = 0.0217; Nurse-only: *p* = 0.0112) during observation, while the proportion of workers found in the nest area in both nurse- and forager-only treatments were significantly higher when compared to controls at time point 2 (Forager-only: *p* = 0.0388; Nurse-only: *p* = 0.0165), 3 (Forager-only: *p* = 0.0179; Nurse-only: *p* = 0.0083), 4 (Forager-only: *p* = 0.0188; Nurse-only: *p* = 0.0085), and 5 (Forager-only: *p* = 0.0247; Nurse-only: *p* = 0.0112) during observation.

Larvae had a significant effect on the proportion of workers found either in the foraging arena (Fig. 1C) and in the nest (Fig. 1D) between the time points 2 and 4 at day 3 (Table S3B). The proportion of workers found in the foraging arena in both nurse-only and forager-only treatments were significantly lower when compared to controls at day 3 at time point 2 (Forager-only: *p* = 0.0023; Nurse-only: *p* = 0.0065), 3 (Forager-only: *p* = 0.0125; Nurse-only: *p* = 0.0047), and 4 (Forager-only: *p* = 0.0113; Nurse-only: *p* = 0.0072) during observation. Conversely, the proportion of workers found in the nest area in both nurse- and forager-only treatments were significantly higher when compared to controls at time point 2 (Forager-only: *p* = 0.0023; Nurse-only: *p* = 0.0065), 3 (Forager-only: *p* = 0.0125; Nurse-only: *p* = 0.0047), and 4 (Forager-only: *p* = 0.0113; Nurse-only: *p* = 0.0072), during observation.

Pupae had a significant effect on the proportion of workers found in the foraging arena (Fig. 1E) and in the nest (Fig. 1F) at time point 4 for day 3 (Table S3B). The proportion of workers found in the foraging arena in forager-only treatments was significantly higher than the proportion of workers found in the foraging arena compared to nurse-only treatments (*p* = 0.0061). Likewise, there were significantly more workers in the nest-area in the nest from nurse-only treatments compared to forager-only treatments (*p* = 0.0061).

### Task reversion analysis

**Figure 2:**
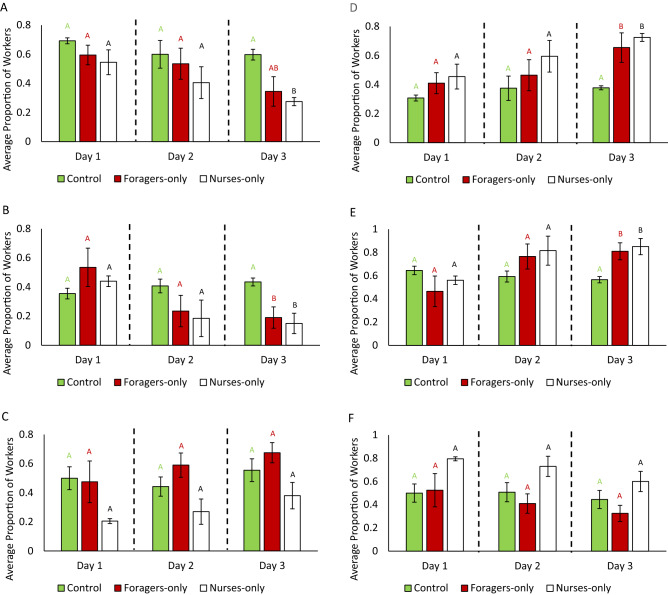
Task reversion analysis: average proportion (± standard error of the mean) of workers in the controls, forager-only treatments, or nurse-only treatments found performing either nursing or foraging behavior during each day of interaction with the different brood types. The proportion of workers found in the foraging arena during the 3 days while in the presence of (**A**) eggs, (**B**) larvae, and (**C**) pupae for each treatment, and the proportion of workers found in the nest during the 3 days while in the presence of (**D**) eggs, (**E**) larvae, and (**F**) pupae for each treatment. Different letters denote statistical differences (*p* < 0.05). Bars represent the standard error of the mean.

The type of brood had a significant effect on the average proportions of workers found in the foraging arena as well as the nest (Fig. 2), but only during day three for workers in the presence of eggs (Fig. 2A) and larvae (Fig. 2B), but not pupae (Fig. 2C; Table S3C).

For day three, the proportion of workers in the presence of eggs found in the foraging arena was significantly lower in nurse-only treatments compared to controls (*p* = 0.015, Fig. 2A), when the proportion of workers found in the nest was significantly higher in both forager-only (*p* = 0.026) and nurse-only (*p* = 0.008) treatments compared to controls (Fig. 2D). In the presence of larvae, both forager-only (*p* = 0.045) and nurse-only (*p* = 0.022) treatments had significantly lower proportions of workers found in the foraging arena compared to controls (Fig. 2B), while also having significantly more workers found within the nest in forager-only (*p* = 0.045) and nurse-only (*p* = 0.022) treatments compared to controls (Fig. 2E). In the presence of pupae, there was no significant difference in the proportions of workers found in either the nest or within the foraging arena among the control, forager-only and nurse-only treatments (Fig. 2C and F).

### Individual worker tracking

Individual workers were tracked as to which behavior they were performing during the observation to discern if the workers that were behaviorally reverting or accelerating were consistently the same, or if they were different every time. There was a significant relationship between the task a worker performed during the first and last observation each day for both the foragers-only treatment with eggs, larvae, and pupae, and the nurses-only treatment with eggs, larvae, and pupae when comparing time point 1 to time point 5 (Table 1).

**Table 1:** Number of workers remaining on task or switching tasks between the observations at time point 1 and time point 5 each day.

Treatment group	Brood	Day	Remained forager	Switched from forager to Nurse	Remained nurse	Switched from nurse to forager	Likelihood ratio Chi^2^	*p value*
Foragers-only	Eggs	1	14	4	15	7	8.761	0.0031
Foragers-only	Eggs	2	17	4	17	2	22.115	< 0.0001
Foragers-only	Eggs	3	9	13	17	1	7.496	0.0062
Foragers-only	Larvae	1	16	4	17	3	18.427	< 0.0001
Foragers-only	Larvae	2	7	0	32	1	31.07	< 0.0001
Foragers-only	Larvae	3	4	0	32	4	14.916	< 0.0001
Foragers-only	Pupae	1	18	3	19	0	37.826	< 0.0001
Foragers-only	Pupae	2	19	4	15	2	21.783	< 0.0001
Foragers-only	Pupae	3	25	1	12	2	30.486	< 0.0001
Nurses-only	Eggs	1	19	0	17	4	25.062	< 0.0001
Nurses-only	Eggs	2	7	12	17	4	1.595	0.2066
Nurses-only	Eggs	3	9	9	22	0	17.7	< 0.0001
Nurses-only	Larvae	1	11	6	19	4	9.597	0.0019
Nurses-only	Larvae	2	5	0	32	3	19.557	< 0.0001
Nurses-only	Larvae	3	4	2	33	1	13.48	0.0002
Nurses-only	Pupae	1	1	3	34	2	1.364	0.2429
Nurses-only	Pupae	2	10	0	29	1	38.285	< 0.0001
Nurses-only	Pupae	3	12	1	19	8	15.585	< 0.0001

The reported values are the added tallies for each category of the 4 replicates. X^2^ values were estimated using the Likelihood Ratio test on these categorical data. *P* values of less than 0.05 were considered significant.

Furthermore, we created a job matrix based on which location workers were found in during the observation period. Being found in the nest attributed a value of 1 to the matrix and achieving a job matrix score of 3 or greater indicated that the worker was found more often in the nest as opposed to the foraging arena. This also allowed us to visualize how the workers were moving and shift in relation to each observation time point (Table S2).

### Quantitative real-time polymerase chain reaction (qRT-PCR)

**Figure 3:**
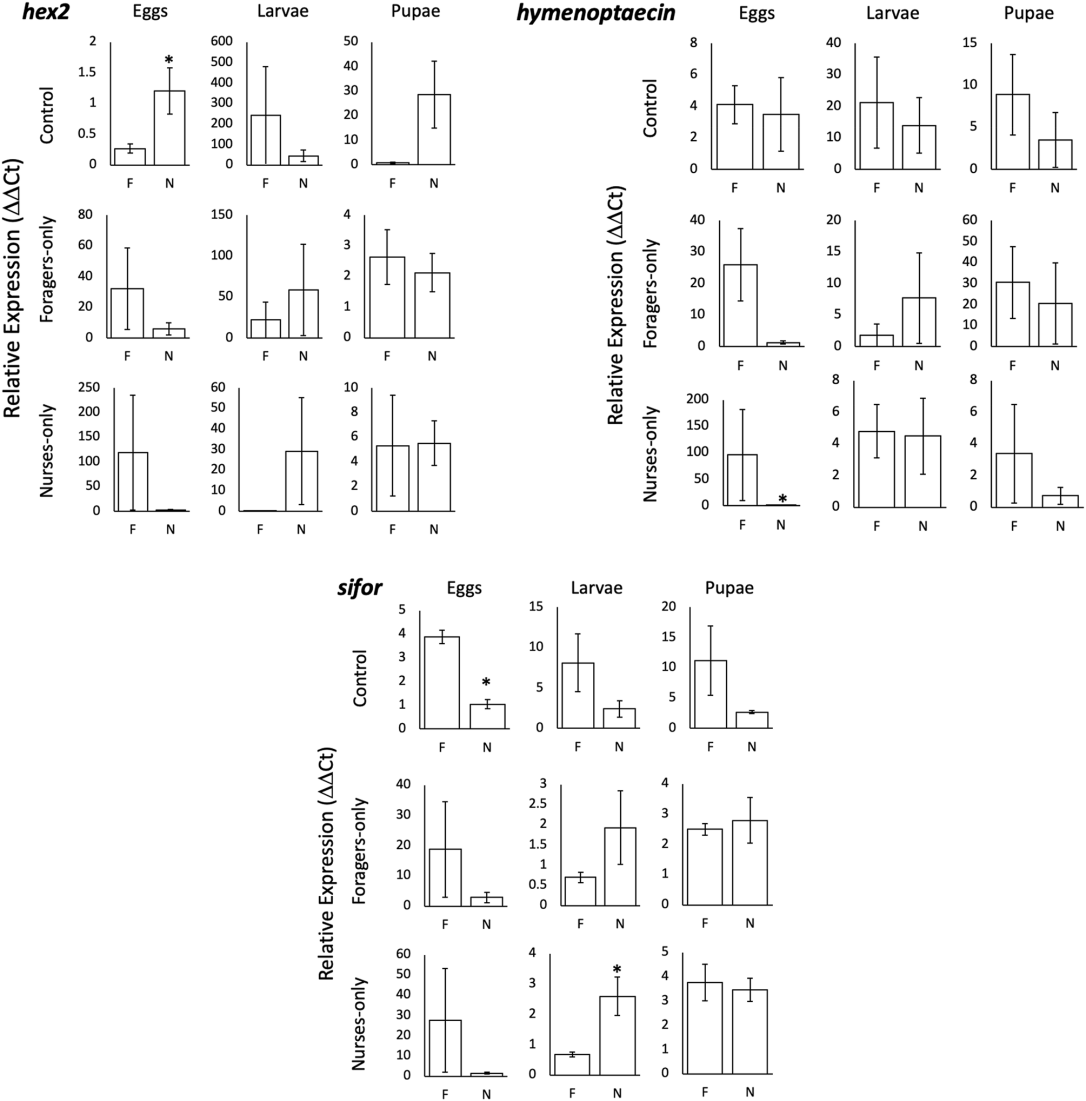
qRT-PCR: relative expression (± standard error of the mean) of *hex2*, *hymenoptaecin* and *sifor* genes in the workers collected from the foraging-arena and nest at the end of the task reversion assay. Data are presented for each treatment and brood type present during the duration of the experiment. ‘F’ and ‘N’ at the bottom of each bar graph describe the workers collected at the end of the experiment performing forager-like behavior or nurse-like behavior. For each condition, n = 4 except for all treatments with pupae for which n = 3, as well as all nurse-only treatment groups found in foraging conditions (n = 3). Gene expression was quantified using RT-qPCR and analyzed using the ΔΔCt method with nurse-only workers found in the nest with eggs as the normalizing group. For each gene, expression level was normalized relative to the housekeeping gene *rp18*. Asterisks denote significance determined by one-way ANOVA (*p* < 0.05).

Workers were collected from the task reversion assay and separated for each treatment and replicate based on the task they were performing at the end of the last time point. With the workers used for qPCR analysis being the same used in the reversion assay, the final counts for replicates regarding the different treatment groups were not consistent as some observations did not have any workers found in either the nest or the foraging arena. qPCR analysis of expression of targeted genes of interest in workers collected from different treatments yielded data which was not significant. However, if data were compared between workers in the foraging arena and the nest for each treatment and brood type independently, some differences were observed (Fig. 3). In the control group, *hex2* showed significant differences in expression in the presence of eggs (Fig. 3, F = 8.97, df = 3, *p* = 0.0242) with nurse-like workers showing higher expression compared to foragers. *sifor* was differentially expressed in the control group in the presence of eggs (Fig. 3, F = 55.05, df = 3, *p* = 0.0003), with forager-like workers having higher expression compared to nurses. *Hymenoptaecin* was differentially expressed in the foragers-only treatment in the presence of eggs. These genes were not differentially expressed between workers in the nest and foraging arena at the end of the task reversion assay in all other treatments.

## Discussion

This study evaluated the role of brood in task behavior of the red imported fire ant, *S. invicta*. We observed that the task performed by workers could be reassigned in as little as 5 min post-worker manipulation in the micro-colony. Indeed, by the first observation in day 1, there were similar proportion of workers performing nest- and foraging-like tasks independently of the task of the worker before worker manipulation. This indicates that shifts in division of labor in this species can be rapid post-colony disturbance. The ability to shift tasks and revert age polyethism allows for an adaptive response to whatever needs the colony might have at a given time^3,17,20,22,70^. Our experiment evaluated medium workers only. It is likely that results could be different if minors or majors were tested, because medium workers show flexibility in task within the colony^51^, and may have a higher propensity towards task reversion in regard to the fixed response threshold.

Overall, the total number of workers in the manipulated colonies was lower than in the control colonies. This setup was chosen to have enough workers performing each task in the controls so there would be no reversion or progression during the experiment. Indeed, on average workers maintained their original task throughout the experiment in the control treatments, half performed nurse-like tasks and half of the workers performed foraging-like tasks. Similar trends were observed in the presence of eggs on days 1 and 2 in the forager-only colonies and in the nurses-only colonies, albeit a tendency to a higher proportion of nurses was observed in these latter colonies. On day 3, there were significant differences in the proportion of workers performing nest-like or foraging-like behaviors when compared to the controls. The higher proportion of workers performing nurse-like behavior could be linked to the observed egg hatching by the end of the experiment. It is possible that five nurses were not enough to care for the eggs and young larvae after egg hatching resulting in a higher proportion of ants performing this behavior. This switch was not observed in the controls in which it is probable that the higher number of ant nurses was enough to care for the progeny. Similar results were observed in the presence of larvae, however, in this case the tendency towards a higher proportion of workers performing nurse-like behaviors was observed in day 2 for both types of manipulated colonies. In the presence of pupae, there was no difference between the control and manipulated colonies. However, there was a tendency for a higher proportion of workers maintaining their initial task. Therefore, the type of brood present changed the proportion of workers which reverted or even accelerated in behavioral task within the micro colonies. Brood within eusocial colonies communicate their needs in order to receive care from adult workers^9,71–75^, thus it would be expected that the developmental phase in which worker attention is most required, the larval stage, would have the greatest impact on task reversion or acceleration in *S. invicta* because of the need for active, continuous care. Moreover, because 4th instar larvae are used by the colony to digest solid proteins^62^, there is an even greater stress on the importance of this particular type of brood within a colony. Pupae seemingly affected the foragers-only treatment groups, which after initiating the experiment immediately had nearly half of the total workers in the nest, while workers in the nurse-only treatment tended to remain in the nest. However, in *S. invicta*, there are currently no documented cases of pupae communicating with adults chemically, and thus would be interesting to investigate how the pupae caused these changes in worker behavior, such as pupal cuticular compounds or acoustics. The eggs used for the bioassays were of indetermined age. Thus, in some cases, there would be 1st instar larvae at the end of the 3-day observations that were seen in the colonies given eggs. While eggs would provide little chemical communication, 1st instar larvae would provide chemical and behavioral communication towards workers. The lack of significance within treatment groups given eggs shows that workers shifted their behavior in such a way that resembled the controls; with more of an even balance of nurses and foragers in groups that were only nurses and foragers respectively.

The workers found either foraging or nursing within the micro-colony setups for each of the different treatment groups were consistent, almost always consisting of the same workers in each observation (Table 1). The plasticity of worker behavior allows them to shift from nurses to foragers as they age^50^. However, the tasks being performed by workers need to be controlled so that all the needs of a colony are being met^19^. It would be genetically and energetically taxing for an organism to constantly shift from a behavioral specialization to another, and thus it would be reasonable to surmise that once worker behavioral roles are determined within a micro-colony setup, that they would remain that way until the status of the colony changes over time.

The expression of the tested genes was not what we had expected in the control groups bar for the micro-colonies in the presence of eggs for *hex2* and *sifor*. The most likely reason for this is that the micro-colonies are a disturbed variation of a normal colony, where there is no queen present as well as a drastically reduced population in adult females. Despite these pitfalls the micro-colony setup was chosen because of the following reasons. First, the difficulties marking a large number of individual fire ants and having said markings remain on the individual^49^. Second, having a queen present would alter the composition of brood within a micro-colony at any given point in the experiment, preventing us from accurately testing the effects of a specific type of brood on worker behavior. Despite this, we still believed it was interesting to observe any differential expression that might be present across treatment groups.

The lack of differential expression of the tested genes across the different treatments as well as the within the different brood types was interesting to observe. While not significant, the gene expression profile was as expected in the controls in the presence of pupae for *hex2* (higher expression in workers in the nest that in the foraging arena), and for *hymenoptaecin* (higher expression on the workers in the foraging arena) as well as for *sifor* in the controls for the presence of the three brood types. While behavioral changes are certainly occurring, depending on the regulation mechanisms, the time required to regulate gene expression to match the behavior observed may be longer than the duration of the assays. For example, if gene expression is regulated by hormones such as juvenile hormone^76^. Though, due to some of the treatment groups having less replicates compared to others, some of the statistical comparisons may not be significant despite them showing a strong trend. Additionally, the large amount of variation observed with these samples may be due to the varied nature of tasks the workers were performing: while we observed a tendency to keep on track, ants were observed switching from nest to foraging arena and vice versa during the time points prior to the sample collection. It is interesting to observe that the expression profiles for *sifor*, *hymenoptaecin*, and *hex2* were similar across the nurses-only and foragers-only treatment groups in the presence of each of the different brood types (Fig. 3). This may indicate that these workers may have transitioned behaviorally from their specialized roles as either foragers or nurses to reserve-like intermediate workers, which typically make up the majority of any *S. invicta* colony^51^. Alternatively, the lack of significant differences between these genes across workers found within the nest and within the foraging arena may be indicative that these genes, while previously shown to be differentially expressed between foragers and nurses, may be more associated with age as opposed to the task workers are performing.

The results from this experiment have the potential for application in the pest management aspect of this invasive organism. First, the biomarkers we used (*hex2, hymenoptaecin,* and *sifor*) could be potential targets for silencing to control *S. invicta* via RNAi. Additionally, we showed that in the presence of certain brood types, particularly eggs and larvae, workers change their behavior based on their colony make up. Thus, finding a way to manipulate the amounts and ratios of these types of brood within a colony or the signals from these brood types recognized by the workers and resulting in worker recruitment for specific tasks could be used to either disrupt major colony function, attract and kill workers, or even manipulate foraging efficiency among workers to be more easily targeted by RNAi bait traps. Whether or not the effect of brood on workers is via a form of chemical or physical communication reveals the potential to have that signal be disrupted, thus depriving not only new generations of workers and reproductives from receiving parental care, but also reducing care towards the fourth-instar larvae, the only individuals within the colony that are able to break down solid proteins.

Overall, division of labor and its regulatory processes are a product of the needs of the colony. This study reveals that the phenomenon of worker task reversion that has been described in other species is present in *S. invicta*. While the presence of brood has been shown in this study to play a role in task reversion, other factors such as how colony genotype affects this process remain unknown. Though we know that age polyethism is present in *S. invicta*, we did not keep track of worker age, and did not observe this behavior in other subcastes such as minors or majors. The effects of different types of brood, and of worker age in the different subcastes should be evaluated in the future to better understanding of division of labor in *S. invicta*. Finally, these results were obtained for polygyne workers, and the effect of brood may shift depending on the colony genotype, thus making a similar experiment performed on monogyne colonies in the future something to investigate. The results from this experiment show that the mechanisms involved in the shift of tasks performed by *S. invicta* workers are complex, and that brood seems to have a prominent effect on behavioral changes. These results also show that while worker behavior is not drastically different when observed within a queenless micro-colony setup, the gene expression of said workers do not reflect what is typically observed within a queenright colony.

## Supplementary Information

Supplementary Table S1.

Supplementary Table S2.

Supplementary Table S3.

## Data Availability

The datasets used and analyzed during the current study are available from either author upon reasonable request.
